# Risk of aortic aneurysm in patients with psoriasis: A systematic review and meta‐analysis of cohort studies

**DOI:** 10.1002/clc.23438

**Published:** 2020-08-05

**Authors:** Xinyu Yu, Xin Feng, Liangtao Xia, Shiyi Cao, Xiang Wei

**Affiliations:** ^1^ Department of Neurosurgery, Union Hospital, Tongji Medical College Huazhong University of Science and Technology Wuhan China; ^2^ Division of Cardiothoracic and Vascular Surgery, Tongji Hospital, Tongji Medical College Huazhong University of Science and Technology Wuhan China; ^3^ School of Public Health, Tongji Medical College Huazhong University of Science and Technology Wuhan China; ^4^ Key Laboratory of Organ Transplantation Ministry of Education Wuhan China; ^5^ NHC Key Laboratory of Organ Transplantation Wuhan China; ^6^ Key Laboratory of Organ Transplantation Chinese Academy of Medical Sciences Wuhan China

**Keywords:** aortic aneurysm, meta‐analysis, psoriasis

## Abstract

**Background:**

The association between psoriasis and the risk of aortic aneurysm is still unclear.

**Hypothesis:**

Patients with psoriasis have a higher risk of aortic aneurysm than healthy individuals.

**Methods:**

PubMed, Embase, and Scopus from inception to 20 July 2019 were searched. We included cohort studies if they reported estimate effects on the risk of aortic aneurysm in patient with psoriasis. We used Newcastle‐Ottawa Scale to evaluate methodology quality of eligible studies. Random‐effect meta‐analyses were used to estimate the overall risk. Subgroup analyses were conducted for analysis of influencing factors.

**Results:**

After a view of 2207 citations, we included three large cohort studies enrolling 5 706 525 participants in this systematic review. Psoriasis patients have an increased risk of development of aortic aneurysm (hazard ratio [HR]: 1.30, 95%confidence intervals [CI], 1.10‐1.55, *I* ^2^ = 53.1%). The risk is not statistically different between patients with severe psoriasis (HR, 1.51, 95%CI, 1.04‐2.19, *I* ^2^ = 40.2%) and patients with mild psoriasis (HR, 1.24, 95%CI, 1.08‐1.42, *I* ^2^ = 24.1%). The risk was not statistically increased in female patients (HR, 1.55, 95%CI, 0.65‐3.72), patients ≥50 years old (HR, 4.05, 95%CI, 0.69‐23.75, *I* ^2^ = 97.3%), and patients with diabetes (HR, 0.97, 95%CI, 0.83‐1.14).

**Conclusions:**

Current evidence from observational studies suggests that psoriasis increases the risk of aortic aneurysm, and screening of aortic aneurysm might be considered among psoriasis patients.

## 1 INTRODUCTION

Psoriasis is a systemic inflammatory disease that affects an estimated 3% of the US adult population.^1^ Patients with psoriasis have an increased prevalence of cardiovascular diseases, including ischemic heart disease,^2^ heart failure,^3^ peripheral vascular disease,^4^ and stroke.^5^ The increased risk of cardiovascular events is believed to be associated with the systematic inflammatory pathophysiological mechanisms of psoriasis.^6^Moreover, aortic vascular inflammation detected by^18^Fluorodeoxyglucose (FDG) positron emission tomography/computed tomography (PET/CT) is associated with psoriasis skin disease severity.^1^, ^7^

Aortic vascular inflammation plays an essential role in the development and progression of aortic aneurysm (AA). Chronic aortic vascular inflammation is believed to lead to destruction of the aortic media and to vascular smooth muscle cell dysfunction as a result of the release of a range of proteolytic enzymes, such as matrix metalloproteinases and cysteine proteases, oxidation‐derived free radicals, cytokines, and related products.^8^ Given these foundations, several studies^9^, ^10^, ^11^ have explored the relative risk of AA in patients with psoriasis. However, these study findings are conflicting, and whether psoriasis increases the risk of AA is still unclear. Thus, we conducted this systematic review to investigate the risk of AA in psoriasis patients.

## 2 METHODS

### 2.1 Search strategy

The systematic review followed the Preferred Reporting Items for Systematic Reviews and Meta‐analyses (PRISMA) guidelines. We conducted a systematic search on PubMed, Scopus, and Embase for relevant full‐text articles published before July 20, 2019. We did not set any language limitation in the literature search. Search terms (displayed in Electronic supplementary material, ESM) included a combination of keywords relating to psoriasis (eg, “psoriatic arthritis”), aortic vascular inflammation (eg, ”vascular inflammation”) and aortic aneurysm (eg, “aorta”, “aortic aneurysm”). To capture other potentially relevant articles, we manually checked the references of the included literature.

### 2.2 Inclusion criteria and study selection

To clarify the association between disease severity of psoriasis and the risk of AA, we included observational studies that satisfied the following criteria: studies reported effect estimates on the risk of AA in patients with psoriasis compared to healthy subjects; psoriasis patients undergoing phototherapy, topical therapy, oral‐systemic medications or biologic agents for psoriasis treatment were eligible; AA of participants could be thoracic AA or abdominal AA; disease severity of psoriasis in participants could be mild to severe.

Conference abstracts were excluded as their full study reports could not be assessed and their scientific rigor had not been peer‐reviewed.^12^ Case reports and case series were excluded for lack of strict study design. Two investigators (Xinyu Yu and Xin Feng) independently screened identified articles to find eligible studies, and the senior investigator (Shiyi Cao) solved discrepancies in study selection.

### 2.3 Data extraction

We extracted the following information from eligible studies: (a) authors, (b) published year, (c) participants, (d) exposure, (e) definition of psoriasis severity, (f) outcomes, (g) controls, (h) follow‐up, (i) unadjusted and adjusted estimates with corresponding 95% confidence interval (CI), (j) adjustment of covariates, and (k) baseline characteristics of study population (age, gender, and comorbidity).

### 2.4 Quality appraisal

We applied the Newcastle‐Ottawa Scale (NOS) to appraise the methodological quality of included studies. The scale is a scoring system covering three perspectives of methodology: selection of study population; comparability; and ascertainment of outcome and exposure. We did not exclude any study on the basis of quality appraisal. Two investigators (Xinyu Yu and Xin Feng) individually assessed the quality of eligible studies, and a senior investigator (Shiyi Cao) solved the discrepancies.

### 2.5 Statistical analyses

Unadjusted and adjusted hazard ratios (HRs) with corresponding 95%CI were extracted from included studies. We used adjusted HRs to reduce the impact of confounding factors on estimate effects. There was obvious heterogeneity in study methodology and definition of psoriasis severity between included articles. Therefore, we just used random‐effect meta‐analysis to evaluate the risk of AA in groups with psoriasis. Statistical heterogeneity was quantified by using the inconsistency index (*I* ^2^) test.^13^ *I* ^2^ values ranged from 0% to 100% and *I* ^2^ < 50% was considered as low heterogeneity, and *I* ^2^ value 50% to 75% as moderate heterogeneity, and *I* ^2^ > 75% as statistically high heterogeneity. When we analyzed the overall risk of AA in patients with psoriasis and the overall HR was not provided, we used fixed‐effect meta‐analysis to calculate overall HR from separate HR. Due to the limited number of included study, we did not conduct a sensitivity analysis. We performed subgroup analyses to find whether other factors (eg, age, gender, and comorbidities) influence the final estimates. We conducted a meta‐regression analysis to test the difference between subgroups. We used Student *t* test to compare the differences in mean values between two groups, and we conducted Chi‐square test to evaluate the significant difference of baseline factors in two groups. All *P* value were two‐tailed and *P* < .05 was set as the significance level. All analyses were conducted in Stata version 14.0 and forest plots were prepared in R version 3.6.1.

## 3 RESULTS

### 3.1 Study characteristics and quality appraisal

**FIGURE 1:**
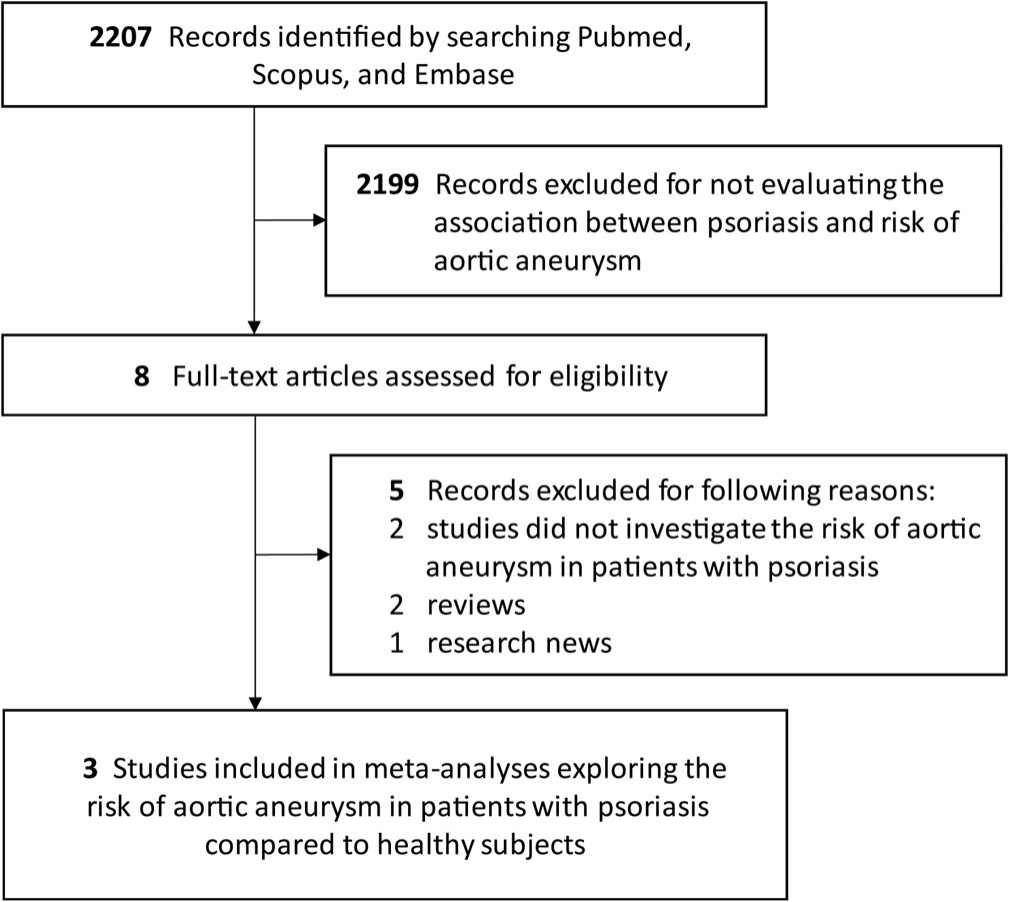
Study screening flowchart

The searching strategy is displayed in Figure 1. In total, 2207 records were identified, and eight articles were assessed for eligibility after screening. Finally, three studies^9^, ^10^, ^11^ with 5 706 525 participants were included in this systematic review. The included studies were scored 7 to 8 points in NOS (Supplementary Table 1). Detailed characteristics of included studies and baseline characteristics of included population were arranged in Table 1 and Supplementary Table 2. Three studies were performed in United States, Denmark, and Taiwan, respectively. Basic features (age, sex, and comorbidity) were significantly different between psoriasis cases and reference population, and we used HRs adjusted by these factors to avoid the interference of them on pooled results.

**TABLE 1:** Characteristics of studies included in the meta‐analysis

Study Author and published Year (location)	Study design	Participants	Exposure	Definition of psoriasis severity	Outcomes	Controls	Mean follow‐up duration	Unadjusted estimate (95% CI) effects of the risk of AA	Adjusted estimate (95% CI) effects of the risk of AA	Adjustment of covariates
Chiu et al. 2016 (Taiwan)^9^	Retrospective population‐based matched cohort study	Taiwan residents aged ≥18 years and without prior AA or aortic dissection in the National Health Insurance Research Database (n = 171 505)	A diagnosis of psoriasis twice by dermatologists during ambulatory visits or inpatient care (ICD‐9 codes 696.0, 696.1696.8)	Severe psoriasis was defined as receiving systemic antipsoriatic therapy, phototherapy, or both at least once during the first 3 years of follow‐up; otherwise, patients were considered to have mild psoriasis	The first ambulatory visit, hospitalization, or surgical procedure for AA, irrespective of whether patients were alive or deceased after the disease	For each case, four healthy subjects matched for age and sex were selected	6.5 years	All cases, HR: 2.07 (1.47‐2.91). Mild psoriasis, HR: 1.91 (1.28‐2.86). Severe psoriasis, HR: 2.58 (1.34‐4.98)	All cases, HR: 1.80 (1.25‐2.61). Mild psoriasis, HR: 1.73 (1.12‐2.65). Severe psoriasis, HR: 2.15 (1.03‐4.51)	Cardiovascular conditions, comorbidities (hypertension, hyperlipidemia, diabetes, chronic kidney disease, atherosclerosis, stroke, bicuspid aortic valve, stenosis of carotid or peripheral artery, rheumatoid arthritis, obesity, alcohol dependence, and tobacco use disorder), and medication use during the preceding year
Khalid et al. 2016 (Denmark)^10^	Retrospective cohort study	All Danish citizens aged ≥18 years and subjects were included in the study on January 1, 1997, or the subsequent day that they reached 18 (n = 5 495 203)	Psoriasis identified by claimed prescriptions of topical vitamin D derivatives (ATC D05AX) or second prescription claims for these agents for persistent medical treatment	Patients with severe psoriasis were identified by hospitalizations for psoriasis (ICD‐10 code L40) or psoriatic arthritis (ICD‐10 codes M070‐M073) and included at the time of their third diagnosis; otherwise, patients were regarded to have mild psoriasis.	The first diagnosis of AAA (ICD‐10 codes DI71.4, DI71.6, DI71.9, DI71.9A, DI79.0, and ICD‐8 code 441)	Participants without psoriasis and AA at the beginning of the follow‐up	NA	NA	All cases, HR: 1.27 (1.11‐1.46). Mild psoriasis, HR: 1.20 (1.03‐1.39). Severe psoriasis, HR: 1.67 (1.21‐2.32)	Age, sex, calendar year, comorbidity (atrial fibrillation, diabetes, hypertension, vascular disease, and thromboembolism), medication, socioeconomic status, and smoking
No et al. 2018 (USA)^11^	Retrospective cohort study	Participants recruited in the Kaiser Permanente Southern California Health Plan (n = 59 487)	A diagnosis of psoriasis (details were not mentioned)	Severe psoriasis was identified by using systemic therapy or phototherapy, and the rest was mild psoriasis	The development of AA (details were not mentioned)	For patients with psoriasis, matched healthy subjects were selected	5.6 years	All cases, HR: 1.17 (0.98‐1.40). Mild psoriasis, HR: 1.21 (1.01‐1.45). Severe psoriasis, HR: 0.89 (0.53‐1.52)	All cases, HR: 1.17 (0.98‐1.40). Mild psoriasis, HR: 1.19 (0.99‐1.43). Severe psoriasis, HR: 1.02 (0.60‐1.73)	Confounding demographic and clinical comorbidities such as obesity, hypertension, diabetes mellitus and dyslipidemia

Abbreviations: AA, aortic aneurysm; ICD, international classification of diseases.

### 3.2 Risk of AA in patients with psoriasis

**FIGURE 2:**
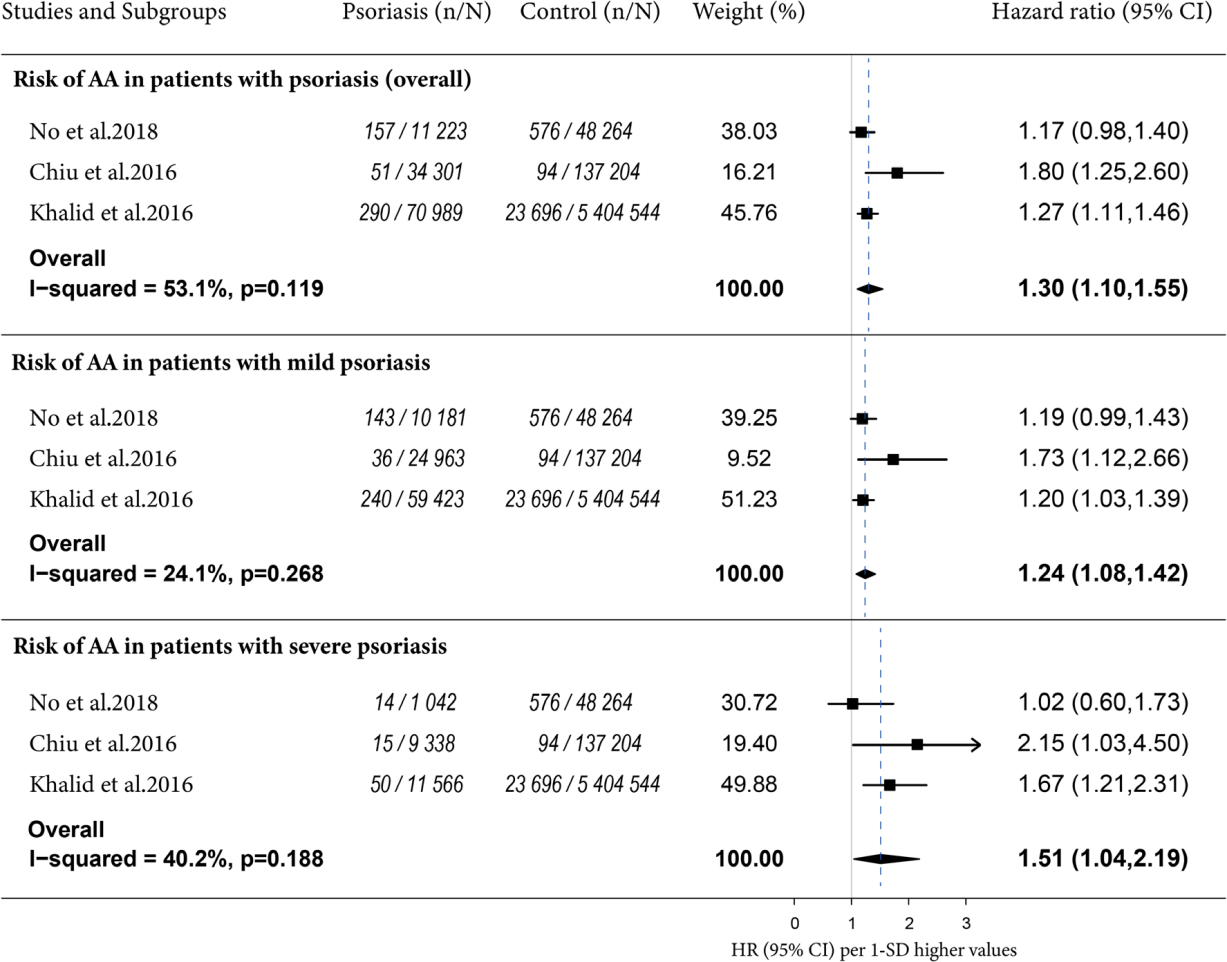
Forest plots of the meta‐analyses for the risk of AA in patients with psoriasis. AA, aortic aneurysm; CI, confidence interval; HR, hazard ratio; n, number of cases of aortic aneurysm; N, number of participants

Forest plots of meta‐analyses were prepared as shown in Figure 2. Three studies with 24 864 cases indicated an HR of 1.30 (95%CI, 1.10‐1.55, *I* ^2^ = 53.1%) for the risk of AA in patients with different degrees of psoriasis. Increased risks of AA were also observed in patients with mild (HR, 1.24, 95%CI, 1.08‐1.42, *I* ^2^ = 24.1%) and severe (HR, 1.51, 95%CI, 1.04‐2.19, *I* ^2^ = 40.2%) psoriasis. There was no significant difference between risks of these two groups (*P* = .278).

### 3.3 Subgroup analysis

According to the subgroup analysis (Table 2), psoriasis could increase the risk of abdominal AA and thoracic AA. However, risks of AA were not significantly increased in female patients (HR, 1.55, 95%CI, 0.65‐3.72), patients ≥50 years old (HR, 4.05, 95%CI, 0.6923.75, *I* ^2^ = 97.3%), and patients with diabetes (HR, 0.97, 95%CI, 0.83‐1.14).

**TABLE 2:** Subgroup analysis

Subgroup	Number of studies	HR	95% confidence interval	*I* ^2^	*P* for heterogeneity
Overall	3	1.30	1.10‐1.55	53.1%	.119
Psoriasis level					
Mild	3	1.24	1.08‐1.42	24.1%	.268
Severe	3	1.51	1.04‐2.19	40.2%	.188
Gender					
Female	1	1.55	0.65‐3.72	—	—
Male	2	2.43	1.53‐3.86	77.7%	.034
Age					
<50 years old	1	2.81	1.15‐6.85	—	—
≥ 50 years old	2	4.05	0.69‐23.75	97.3%	**<**.001
Subtypes of AA					
Abdominal AA	2	1.29	1.14‐1.47	0.0%	.497
Thoracic AA	1	3.03	2.10‐4.37	—	—
Comorbidities					
With hypertension	2	2.09	1.11‐3.95	81.1%	.021
Without hypertension	1	1.23	1.14‐1.34	—	—
With diabetes	2	0.97	0.83‐1.14	0.0%	.721
Without diabetes	1	1.86	1.28‐2.87	—	—
With dyslipidemia	2	1.92	1.53‐2.41	0.0%	.737
Without dyslipidemia	1	1.69	1.15‐2.48	—	—

Abbreviations: AA, aortic aneurysm; HR, hazard ratio.

## 4 DISCUSSION

This is the first systematic review and meta‐analysis to estimate the risk of AA in patients with psoriasis considering the effect of disease severity of psoriasis on the risks. It showed that psoriasis patients are more susceptible to AAs compared to the general population, and the risk does not increase with the severity of psoriasis. Moreover, the association was not significant in female patients, patients ≥50 years old and patients with diabetes.

There was low to moderate statistical heterogeneity within meta‐analyses and subgroup analyses. Definitions and inclusion criteria of exposure and outcome were slightly different between included studies (see Supplementary Table 2), and this led to inevitable clinical heterogeneity. Subgroup analyses showed age, gender, and comorbidity with diabetes affect risks of psoriasis. Diabetes is associated with a reduced incidence of AA,^14^, ^15^ and it decreased the risk of psoriasis in psoriasis patients in this research.

Psoriasis is an immune‐mediated inflammatory disease associated with cardiometabolic comorbidities.^5^, ^16^ Studies have confirmed that psoriasis increases the risk of subclinical cardiovascular disease, as evidenced by higher coronary artery calcium^17^ and an elevated burden of coronary artery disease.^1^, ^18^ Elevated blood inflammatory biomarkers in patients with psoriasis indicate a moderate role of systemic inflammation in pathophysiology of psoriasis.^19^ To identify concrete inflammatory lesions, researches detected metabolic activity in vessels and other tissues using^18^FDG‐PET/CT,^1^, ^7^, ^20^, ^21^, ^22^ and researchers observed significant increase in aortic inflammation in psoriasis patients. Currently, inflammation was considered to have a crucial pathogenic role in the development and progression of AA.^8^ Psoriasis is an immune‐mediated genetic disease^23^ and AA could be an acquired disorder. Therefore, natural course of psoriasis might promote the development of AA. Otherwise, hemodynamic factors and aortic stiffening could also contribute to AA development.^10^, ^24^ In patients with psoriasis, increased arterial stiffness is presented^25^ and it is associated with systemic inflammation.^26^ Aortic stiffening leads to axial stress which then induces and augments processes necessary for AA growth, such as inflammation and aortic wall remodeling.^24^

From a preventive point of view, AA screening is strongly suggested recently.^27^ In light of present findings, AA screening might be conducted among patients with psoriasis, especially in males and people younger than 50 years old. Severe skin inflammation of psoriasis indicates elevated aortic inflammation,^1^ thus increasing the probability of the development of AA. AA screening of psoriasis with higher psoriasis area severity index score could be more effective and economical. From a therapeutic perspective, anti‐inflammatory agents, such as tumor necrosis factor‐α (TNF‐α) antagonist^28^, ^29^ could reduce vascular inflammation and improve endothelial function. Currently, no effective drug therapy is available for AA,^30^ and anti‐inflammatory agents may become potential drugs limiting the development and progression of AA.

When interpreting results of this systemic review, several strengths and limitations should be considered. We conducted subgroup analyses to test the robustness of the results, and we found that age, gender, and comorbidities affect the association. However, we failed to conduct sensitivity analyses and a meta‐regression of influencing factors for lack of enough studies. There is moderate heterogeneity in meta‐analyses and subgroup analyses, and results of this systematic review should be interpreted carefully.

## 5 CONCLUSION

Our findings indicate a higher incidence of AA in patients with psoriasis compared to the general population, and the risk does not increase with the disease severity of psoriasis. Whether AA screening is effective and feasible in patients with psoriasis still requires further research.

## CONFLICT OF INTEREST

The authors declare no potential conflict of interest.

## Supporting information


**Supplementary Table 1** Newcastle‐Ottawa scale for assessing the methodological quality of included studies
**Supplementary Table 2.** Baseline characteristics of the study population
**PRISMA check list**
