# Supplementary material for: Risk of aortic aneurysm in patients with psoriasis: A systematic review and meta‐analysis of cohort studies
Source: Clin Cardiol. 2020 Aug 5;43(11):1266–72. doi: 10.1002/clc.23438 (PMC7661651; doi:10.1002/clc.23438)
Supplement: Supplementary file 1 — Supplementary Table 1 Newcastle‐Ottawa scale for assessing the methodological quality of included studies Supplementary Table 2. Baseline characteristics of the study population PRISMA check list [file CLC-43-1266-s001.docx]

**Risk of aortic aneurysm in patients with psoriasis: a systematic review and meta-analysis of cohort studies**

Xinyu Yu^a#^, Xin Feng^a#^, Liangtao Xia^a^, Shiyi Cao^b^*, Xiang Wei^a,c,d,e^ *.

a. Division of Cardiothoracic and Vascular Surgery, Tongji Hospital, Tongji Medical College, Huazhong University of Science and Technology, Wuhan 430030, China

b. School of Public Health, Tongji Medical College, Huazhong University of Science and Technology, Wuhan, Hubei, China

c. Key Laboratory of Organ Transplantation, Ministry of Education

d. NHC Key Laboratory of Organ Transplantation

e. Key Laboratory of Organ Transplantation, Chinese Academy of Medical Sciences

# Xinyu Yu and Xin Feng contributed equally to this research.

***Corresponding author:**

Shi-yi Cao: [caoshiyi@hust.edu.cn](mailto:caoshiyi@hust.edu.cn)

Address: No. 13 Hangkong Road, Wuhan 430030, China

Telephone and fax numbers: +86-027-83693756

Or

Xiang Wei：[xiangwei@tjh.tjmu.edu.cn](mailto:xiangwei@tjh.tjmu.edu.cn)

Address: No. 1095 Jiefang Avenue, Wuhan 430030, China

Telephone and fax numbers: +86-027-83663394

**Search strategy:**

1. Pubmed

(aorta OR aortitis OR aortic stenosis OR aortic dissection OR aortic aneurysm OR aortic inflammation OR aortic elasticity OR aortic stiffness OR arterial stiffness OR vascular inflammation OR systemic inflammation OR aortopathy) AND (psoriasis OR psoriatic arthritis)

1. Scopus

(TITLE-ABS-KEY ((aorta OR aortitis OR "aortic stenosis" OR "aortic dissection" OR "aortic aneurysm" OR "aortic inflammation" OR "aortic elasticity" OR "aortic stiffness" OR "arterial stiffness" OR "vascular inflammation" OR "systemic inflammation" OR aortopathy)) AND TITLE-ABS-KEY ((psoriasis OR "psoriatic arthritis")))

1. Embase

(aorta OR aortitis OR 'aortic stenosis' OR 'aortic dissection' OR 'aortic aneurysm' OR 'aortic inflammation' OR 'aortic elasticity' OR 'aortic stiffness' OR 'arterial stiffness' OR 'vascular inflammation' OR 'systemic inflammation' OR aortopathy) AND (psoriasis OR 'psoriatic arthritis')

Supplementary table 1. Newcastle-Ottawa scale for assessing the methodological quality of included studies

| Study  (Pubmed ID) | Selection | | | | Comparability | Outcome | | | Score |
| --- | --- | --- | --- | --- | --- | --- | --- | --- | --- |
|  | Typical  cohort | Rational selection of non-exposed cohort | Ascertainment  Of exposure | Demonstration that outcome of interest was not present at beginning | Comparable cohorts | Assessment of  outcome | Follow up is long enough for outcome to occur | Adequacy of follow up of cohorts |  |
| Chiu et al. 2016 (27473449) | ★ | ★ | ★ | ★ | ★★ | ★ | ★ |  | 8 |
| Khalid et al. 2016  (27079879) | ★ | ★ | ★ | ★ | ★ | ★ | ★ |  | 7 |
| No et al. 2018  (28758265) | ★ | ★ | ★ |  | ★★ | ★ | ★ |  | 7 |
|  |  |  |  |  |  |  |  |  |  |

Supplementary table 2. Baseline characteristics of the study population

| Characteristic | Reference Population  ( n=5,590,012) | Psoriasis cases  ( n= 116,513 ) | P value |
| --- | --- | --- | --- |
| Age (years), mean (SD) | 41.1 (19.6) | 44.7 (16.3) | ＜0.001 |
| Sex, *n* (%) |  |  | ＜0.001 |
| Male | 2,766,943 (49.5%) | 59360 (50.9%) |  |
| Female  Comorbidity, *n* (%) | 2,823,069 (50.5%) | 57153 (49.1%) |  |
| Hypertension | 192,000 (3.4%) | 7,560 (6.4%) | ＜0.001 |
| Vascular disease | 47,092 (0.8%) | 3,279 (2.8%) | ＜0.001 |
| Diabetes mellitus | 101,026 (1.8%) | 6,969 (6.0%) | ＜0.001 |
|  |  |  |  |

Abbreviations: SD, standard deviation.

| **Section/topic** | **#** | **Checklist item** | **Reported on page #** |
| --- | --- | --- | --- |
| **TITLE** | | |  |
| Title | 1 | Identify the report as a systematic review, meta-analysis, or both. | 1 |
| **ABSTRACT** | | |  |
| Structured summary | 2 | Provide a structured summary including, as applicable: background; objectives; data sources; study eligibility criteria, participants, and interventions; study appraisal and synthesis methods; results; limitations; conclusions and implications of key findings; systematic review registration number. | 2 |
| **INTRODUCTION** | | |  |
| Rationale | 3 | Describe the rationale for the review in the context of what is already known. | 3 |
| Objectives | 4 | Provide an explicit statement of questions being addressed with reference to participants, interventions, comparisons, outcomes, and study design (PICOS). | 3 |
| **METHODS** | | |  |
| Protocol and registration | 5 | Indicate if a review protocol exists, if and where it can be accessed (e.g., Web address), and, if available, provide registration information including registration number. | 3 |
| Eligibility criteria | 6 | Specify study characteristics (e.g., PICOS, length of follow-up) and report characteristics (e.g., years considered, language, publication status) used as criteria for eligibility, giving rationale. | 4 |
| Information sources | 7 | Describe all information sources (e.g., databases with dates of coverage, contact with study authors to identify additional studies) in the search and date last searched. | 3 |
| Search | 8 | Present full electronic search strategy for at least one database, including any limits used, such that it could be repeated. | 4 |
| Study selection | 9 | State the process for selecting studies (i.e., screening, eligibility, included in systematic review, and, if applicable, included in the meta-analysis). | 4 |
| Data collection process | 10 | Describe method of data extraction from reports (e.g., piloted forms, independently, in duplicate) and any processes for obtaining and confirming data from investigators. | 4 |
| Data items | 11 | List and define all variables for which data were sought (e.g., PICOS, funding sources) and any assumptions and simplifications made. | 4 |
| Risk of bias in individual studies | 12 | Describe methods used for assessing risk of bias of individual studies (including specification of whether this was done at the study or outcome level), and how this information is to be used in any data synthesis. | 4 |
| Summary measures | 13 | State the principal summary measures (e.g., risk ratio, difference in means). | 4-5 |
| Synthesis of results | 14 | Describe the methods of handling data and combining results of studies, if done, including measures of consistency (e.g., I^2^) for each meta-analysis. | 5 |

Page 1 of 2

| **Section/topic** | **#** | **Checklist item** | **Reported on page #** |
| --- | --- | --- | --- |
| Risk of bias across studies | 15 | Specify any assessment of risk of bias that may affect the cumulative evidence (e.g., publication bias, selective reporting within studies). | 5 |
| Additional analyses | 16 | Describe methods of additional analyses (e.g., sensitivity or subgroup analyses, meta-regression), if done, indicating which were pre-specified. | 5 |
| **RESULTS** | | |  |
| Study selection | 17 | Give numbers of studies screened, assessed for eligibility, and included in the review, with reasons for exclusions at each stage, ideally with a flow diagram. | 5-6, Figure 1 |
| Study characteristics | 18 | For each study, present characteristics for which data were extracted (e.g., study size, PICOS, follow-up period) and provide the citations. | 5-6,  Table1-2 |
| Risk of bias within studies | 19 | Present data on risk of bias of each study and, if available, any outcome level assessment (see item 12). | 5, ESM |
| Results of individual studies | 20 | For all outcomes considered (benefits or harms), present, for each study: (a) simple summary data for each intervention group (b) effect estimates and confidence intervals, ideally with a forest plot. | Figure 2 |
| Synthesis of results | 21 | Present results of each meta-analysis done, including confidence intervals and measures of consistency. | 6 |
| Risk of bias across studies | 22 | Present results of any assessment of risk of bias across studies (see Item 15). | 6 |
| Additional analysis | 23 | Give results of additional analyses, if done (e.g., sensitivity or subgroup analyses, meta-regression [see Item 16]). | 6 |
| **DISCUSSION** | | |  |
| Summary of evidence | 24 | Summarize the main findings including the strength of evidence for each main outcome; consider their relevance to key groups (e.g., healthcare providers, users, and policy makers). | 6 |
| Limitations | 25 | Discuss limitations at study and outcome level (e.g., risk of bias), and at review-level (e.g., incomplete retrieval of identified research, reporting bias). | 8 |
| Conclusions | 26 | Provide a general interpretation of the results in the context of other evidence, and implications for future research. | 8 |
| **FUNDING** | | |  |
| Funding | 27 | Describe sources of funding for the systematic review and other support (e.g., supply of data); role of funders for the systematic review. | 8 |

Page 2 of 2
